# Utilization of soft pistachio hulls in Japanese quail diets for enhanced egg quality and yolk pigmentation

**DOI:** 10.7717/peerj.20204

**Published:** 2025-11-21

**Authors:** Mehmet Çetin

**Affiliations:** Department of Animal Science, Faculty of Agriculture, Harran University, Haliliye, Şanlıurfa, Turkey

**Keywords:** Pistachio hull, Egg quality, Yolk pigmentation, Agro-residues, Laying Quail

## Abstract

**Background:**

Japanese quail (*Coturnix coturnix japonica*) offer rapid growth, early maturity and excellent feed conversion, but feed cost accounts for ∼70% of production expenses. This study evaluated soft pistachio hull (PH), an abundant agro-industrial by-product, as a low-cost dietary ingredient in laying quails.

**Methods:**

Ninety-six female quails, seven weeks old age, were randomly distributed to four dietary groups (24 birds each; three replicates of eight birds). The treatment diets were based on a standard layer feed, with graded PH inclusion of 0%, 2%, 4%, and 6%, administered over a for five-week period. Parameters measured included live weight, feed intake, egg production rate, egg weight, feed conversion ratio, and a range of egg quality indicators, such as shape index, shell thickness, Haugh unit, albumen and yolk indices, and yolk color score, assessed at weeks 7 and 11.

**Results:**

Final live weight followed a cubic trend (*P* = 0.05), with the 2% and 6% PH groups (356.5 g and 350.6 g, respectively) exceeding the control (333.9 g), while 4% PH yielded intermediate values. Although differences in egg production were not statistically conclusive, the 4% PH group showed the highest output (76.4%). Feed intake increased by 7.3% in birds fed 4% PH group (*P* < 0.05), with feed conversion efficiency slightly reduced in noth 2% and 6% PH treatments. Egg shape index improved significantly at 4% PH (+3.3%, *P* < 0.01), whereas shell thickness, albumen index and Haugh unit remained unchanged (*P* > 0.05). Yolk color score exhibited strong linear, quadratic and cubic increases (*P* < 0.01), with the 6% PH group scoring 11.43 (+38.9% *vs.* 8.23 control).

**Conclusion:**

Pistachio hulls can be incorporated into laying quail diets up to 6% without adverse effects on egg quality. The 4% inclusion level appears optimal for egg shape and pruductivity, while 6% maximizes yolk coloration. Nonetheless, the observed decline in feed efficiency at some levels suggests that careful ration balancing is essential. The use of PH represents a promising route for upcycling agro-residues into functional feed components with added economic and nutritional value.

## Introduction

The Japanese quail (*Coturnix coturnix japonica*) stands out in the poultry sector for its rapid growth, precocious laying behaviour, and eficient utilization of feed. Typically reaching slaughterweight within five weeks and reproductive maturity by the sixth week, quail farming offers the potential for intensive, high-turnover production systems (Arthur & Bejaei, 2017). While once primarily regarded as a game bird, the Japanese quail now contributes a meaningful share to the global table egg market, estimated at roughly 10% of worldwide egg output (Lukanov, 2019), and accouts for approximatelly 11.8% of total commercial poultry stock (Minvielle, 2004). Although their mean production share remains modest (∼0.2%), their adaptability, reproductive efficiency, and minimla spatial requirements make quail an increasingly attractive species for both smallholder and large-scale producers (Business Research Insights, 2024). Their hardiness and tolerance to environmental variability, including fluctuations in ambient temperature fluctuations and exposure to common avian pathogens, further enhance their suitability for diverse production systems by lowering mortality and veterinary intervention rates (Lima, Morais & Pereira, 2023).

Nutritionally, are valued as nutrient-dense functional foods, offering complete proteins,essential vitamins (A, D, E, and B-complex), and trace minerals such as iron, zinc, and selenium. additionally, the presence of bioactive peptides and immunoglobulins, and antioxidants contributes to their immunomodulatory and health-promoting properties (Nowaczewski et al., 2013; Kovacs-Nolan, Phillips & Mine, 2005; Saeed et al., 2025).

Although quail meat and eggs are nutritionally valuable, the cost of feed remains the dominant factor in production expenses, accounting for nearly 70% of the overall operational budget in quail farming (Mnisi et al., 2021). The reliance on conventional feedstuffs like maize and soybean meal, subject to supply-chain fluctuations and competition with human nutrition and biofuel sectors, necessitates the exploration of alternative feed ingredients Recent studies have investigated the incorporation of agro-industrial residues such as fruit processing waste, oilseed cakes, and cereal by-products into monogastric diets. These materials often provide fermentable fiber, Residual energy, and functional compunds that can support productive performance while reducing environmental load (Georganas et al., 2023).

Pistachio hulls, a primary by-product of pistachio nut processing, are produced in significant volumes in countries like Turkiye, which alone generates over 200,000 tons annually.

Characterized by their fibrous matrix, residual lipid content, and polyphenolic antioxidants, pistachio hulls show potential as a feed component. Their chemical composition shares similarities withlow-grade cereal brans, and includes bioactive compounds that may influence hysiological responses and product quality in poultry (Mahmoud et al., 2024). However, to date no studies have evaluated their inclusion in laying quail diets, nor assessed potential impacts on egg production metrics, internal quality parameters, or shell characteristics.

The present study evaluates the effects of graded levels (0%, 2%, 4%, and 6%) of pistachio hulls in the diets of laying Japanese quails during the 7–11 week laying period, with a particular emphasis on egg production performance and yolk pigmentation. The working hypothesis is that moderate dietary supplemntation with pistachio hulls (especially 2–4%) could improve yolk pigmentation and maintain egg production by leveraging the bioactive and pigment-rich profile of the hulls. Conversely, higher inclusion rates (*e.g.*, 6%) may compromise nutrient digestibility due to excessive fiber or tannin content (Ghasemi, Zarei & Torki, 2014; Viveros et al., 2011). As a whole, this research aims to support circular agriculture by integrating agricultural waste into quail nutrition, contributing to both environmental sustainability and cost-effective egg production practices.

## Materials and Methods

The experimental phase was carried out between November and December 2021 at the Poultry Research Unit of the Faculty of Veterinary Medicine, Harran University, located in Şanlıurfa, southeastern Turkiye (37°9′ 32.9″N, 38°47′ 48.8″E). The experimental environment was designed to meet the physiological needs of the quails, with stable temperature (24 °C), a 16-hour light and 8-hour dark photoperiod, and strict hygiene. Birds had ad libitum access to feed and water, and were monitored daily throughout the trial. Routine procedures such as egg collection, feeding, and watering were performed regularly. No adverse events or clinical abnormalities were observed, and all procedures were completed as planned without requiring removal of any animal.

### Animal material and experimental groups

This study was designed to evaluate the effects of dietary inclusion of fresh pistachio hull (PH) on egg quality and yolk pigmentation in Japanese quails. The key variables used to evaluate the study outcomes were egg production, yolk pigmentation score, and egg weight, which are commonly used to assess egg quality in laying quail. Ethical approval for all experimental procedures was obtained from the Local Animal Ethics Committee of Harran University (Approval Date: April 24, 2021; Document No: 32561).

The sample size per group was determiend based on previous studies with similar experimental designs and field conditions (Özentürk & Yıldız, 2020; Saraçoğlu et al., 2025).

A total of 96 seven-week-old female Japanese quails (*Coturnix coturnix japonica*) were randomly assigned to four dietary treatments: a control group receiving a basal diet and three treatment groups supplemented with 2%, 4%, or 6% PH on a dry matter basis. Each treatment group comprised three replicates of eight birds (*n* = 24 per treatment), housed in standard 6-tier layer cages (45  × 97  × 20 cm). Before group allocation, birds were individually weighed to ensure uniform distribution. A completely randomized design was implemented to assign diets to the treatment groups. Caretakers responsible for feed provision and husbandry were aware of the group allocations for logistical reasons; however, individuals conducting egg quality assessments (*e.g.*, egg weight, yolk color, shell strength) were blinded to treatment assignments to reduce potential observer bias. Data entry and statistical analyses were carried out by the lead investigator, who had access to treatment codes during the analysis phase.

The trial was conducted over a five-week period, corresponding to the early laying phase (7–11 weeks of age). Throughout the experiment, birds had ad libitum access to feed and fresh water. Routine management procedures such as feed replenishment, egg collection, and cage cleaning were performed daily by trained personnel. No mortality or adverse health events occurred during the study.

The quails used in this study originated from fertilized eggs supplied by the Faculty of Veterinary Medicine at Siirt University (Siirt Province, Turkey). These eggs were incubated and hatched at the Harran University Faculty of Agriculture using standard commercial incubation equipment. The hatched chicks were initially reared under controlled conditions and participated in a separate feeding performance trial during their growth phase (0–6 weeks of age). The results of earlier trial have been published (Çetin & Apaydın, 2024), whereas the current study evaluated the same animals during the subsequent laying phase, focusing on distinct outcome parameters such as egg production and yolk pigmentation.

All animals were of standard genetic background and were not subjected to any genetic modification, invasive procedures, or pharmacological treatments; birds were handled gently and only during routine tasks such as weighing and egg collection. Environmental parameters such as temperature, humidity, ventilation, and lighting were optimally regulated to support bird welfare and a stable photoperiod.

Preventive measures were also taken to control pests and rodents. Birds were monitored twice daily for clinical signs of distress, including lethargy, abnormal posture, feather loss, reduced feed intake, or aggressive behavior. All animals remained clinically healthy throughout the study, and no adverse clinical signs or unexpected events were observed. Had any of these signs appeared, affected individuals would have been removed and treated or humanely euthanized according to the guidelines of the American Veterinary Medical Association (American Veterinary Medical Association (AVMA), 2020). However, no such cases occurred. At the end of the experiment, surviving birds were transferred to the AREB facility for egg production.

### Feed material and pistachio hull composition

The basal layer diet used in this study was obtained from a commercial manufacturer and formulated to meet the nutritional needs of laying quails, containing 18.6% crude protein, 3.7% crude fiber, 3.4% crude fat, 12.2% ash, 3.7% calcium, and 0.7% phosphorus.

Pistachio hulls, a fibrous by-product of pistachio nut processing, were sourced from a commercial facility in Şanlıurfa and processed as previously described by Çetin & Apaydın (2024), including drying at 60 °C and grinding to two  mm particle size. Based on prior literature and preliminary observations, PH was incorporated into diets at 2%, 4%, and 6% inclusion levels (on a dry matter basis). These inclusion levels were chosen based on previous literature and preliminary palatability observations (Kohanali et al., 2022; Garrido et al., 2024). Higher levels were not tested due to concerns about potential negative effects on feed intake and nutrient digestibility. All diets were prepared in mash form by thoroughly mixing the ground PH with the commercial layer feed to ensure homogeneity and prevent selective feeding.

The proximate composition of the pistachio hulls, including dry matter, crude protein, crude fiber, fat, ash, and nitrogen-free extract, was determined according to AOAC International (2019) protocols. Metabolizable energy was estimated using validated regression-based formulas (Wiseman et al., 1987). Çetin & Apaydın (2024) reported that PH is rich in fermentable carbohydrates and offer moderate protein and energy levels, making it a viable feed ingredient in poultry diets.

### Methods

Throughout the experiment, various performance and egg quality parameters were recorded.

Individual live body weights (g) of all quails were measured at the beginning (week 7) and at the end (week 11) of the experiment using a precision digital scale (±0.1 g), ensuring consistency in timing (morning before feeding) and handling to minimize stress and measurement variability. Feed intake (g/week) was recorded for each group, and daily egg weights (g/day) were measured using an electronic scale with 0.001 g precision. Weekly feed intake was calculated by subtracting the weight of residual feed (including both uneaten and spilled feed) from the total feed offered. Although feed waste was not expressed as a separate percentage, this approach provided a reliable estimate of actual consumption under ad libitum feeding conditions. Egg yield (%) was calculated by dividing the total number of eggs collected daily by the number of hens in each group. The feed conversion ratio (kg) was expressed as the amount of feed consumed to produce one dozen eggs. Egg mass (g/bird/day) was calculated by multiplying egg production (%) by average egg weight (g) and dividing by 100 (Salajegheh et al., 2012).

The sample size (n) for measurements of live weight, feed intake, egg weight, egg production, and feed conversion ratio was 24 birds per experimental group. For egg quality parameters, including egg shape index, albumen index, yolk index, yolk color, yolk weight, Haugh unit, shell thickness, and shell weight, a sample size of 30 eggs per group was used. Eggs accumulated in each cage were collected during the 7th and 11th weeks of the experiment for quality assessment. These samples were stored at room temperature for 24 h prior to analysis. Each egg was first weighed, and then its length and width were measured using a digital caliper with a precision of 0.01 mm to calculate the shape index using the Eq. (1):

(1) \begin{eqnarray*}\text{Shape Index}~(\%)=(\text{Egg width}/\text{Egg length})\times 100.\end{eqnarray*}

Following dimensional measurements, the eggs were broken onto a glass table and left to settle for 10 min before further evaluation. Albumen height and yolk height (mm) were measured using a micrometer. The length and width of the albumen and the diameter of the yolk were also recorded to calculate albumen and yolk indices. Yolk color was determined using the Roche Colour Fan, which provides a standardized scale from 1 to 15. The Haugh unit (HU), an indicator of egg freshness, was calculated using the Eq. (2) (Rafieian-Naeini et al., 2023).

(2) \begin{eqnarray*}\text{HU}=100\times \text{log}(\text{H}+7.57-1.7\times {\text{W}}^{0.37})\end{eqnarray*}

where *H* is albumen height (mm) and *W* is egg weight (g). The albumen index was calculated by dividing the albumen height by the average diameter and multiplying by 100, as shown in Eq. (3):

(3) \begin{eqnarray*}\text{Albumen Index}~(\%)=(\text{Albumen height}/\text{Average diameter})\times 100.\end{eqnarray*}

Similarly, the yolk index was calculated by dividing the yolk height by the average diameter and multiplying by 100, as indicated in Eq. (4):

(4) \begin{eqnarray*}\text{Yolk Index}~(\%)=(\text{Yolk height}/\text{Average diameter})\times 100.\end{eqnarray*}

For shell quality assessment, shell thickness was measured at the pointed, broad, and blunt ends of each egg using a micrometer, and the average value was recorded. After drying for 24 h, shell weights were determined by weighing the dried shells. All procedures related to egg quality measurements were conducted following the method described by Özentürk & Yıldız (2020).

### Statistical analysis

Data were analyzed under a completely randomized design. All tests were two-sided with *α* = 0.05 and performed in SPSS v22.0 (IBM Corp.). Group differences were evaluated with a one-way analysis of variance (ANOVA) using the model in Eq. (5).

(5) \begin{eqnarray*}{Y}_{ij}=\mu +{\tau }_{i}+{}_{ij}\end{eqnarray*}

where *Y*_*ij*_ is the observed response for the jth treatment (PH dose), *μ* is the overall mean, *τ*_*i*_ is the fixed effect, and *ɛ*_*ij*_ are independent errors with *ɛ*_*ij*_ ∼ N(0, *σ*^2^).

Consistent with ANOVA theory, the model residuals were assessed rather than the raw variables. For each trait, studentized residuals were obtained and normality was evaluated with Shapiro–Wilk test (accompanied by Q-Q plots) and homogeneity of variances with Levene’s tests (median-centered). Diagnostic results are summarized in the Supplementary File. In brief, residual normality and homoscedasticity were satisfied for most endpoints; where residual normality showed minor departures, the inference was verified with a Kruskal–Wallis sensitivity analysis (conclusions were unchanged). When assumptions held, the ANOVA results were interpreted and Duncan’s multiple range test was conducted for *post-hoc* comparisons.

To characterize dose–response relationships, orthogonal polynomial contrasts were fitted (linear, quadratic, and cubic), partitioning the treatment sum of squares into independent components that capture potential stimulation, inhibition, or compensation phases.

## Results

This study evaluated the impact of graded PH inclusion on quail productivity and egg quality across an 11-week laying cycle. A dual focus was maintained on performance metrics, live weight dynamics, egg production efficiency, feed utilization, and feed conversion ratio (FCR) and comprehensive egg quality parameters, encompassing structural (shape index, shell integrity), biochemical (albumen and yolk indices), and pigmentation traits. The results reveal distinct dose-dependent patterns in performance outcomes and egg characteristics, with PH supplementation eliciting nonlinear responses across key metrics. Data are presented in two primary categories: Performance Characteristics (Table 1) and Quality Characteristics of Quail Eggs (Table 2).

**Table 1:** Effect of pistachio hull (PH) doses on live weight, egg weight, egg yield, egg mass, feed consumption and feed conversion ratio.

**Traits**	**Control**	**PH 2%**	**PH 4%**	**PH 6%**	**L**	**Q**	**C**
Beginning LW, g	315.2 ± 35.0	328.9 ± 41.9	322.9 ± 38.2	332.8 ± 32.2	*p* = 0.144	*p* = 0.333	*p* = 0.331
Final LW, g	333.9^ab^ ± 45.6	356.5^a^ ± 32.6	326.1^b^ ± 42.4	350.6^a^ ± 31.8	*p* = 0.589	*p* = 0.859	*P* = 0.026
Egg weight, g	12.57^c^ ± 0.6	13.05^ab^ ± 0.7	12.91^b^ ± 0.6	13.33^a^ ± 0.7	*P* = 0.000	*P* = 0.000	*P* = 0.000
Egg yield, %	74.9 ± 2.4	66.0 ± 3.6	76.4 ± 4.4	71.7 ± 5.7	*p* = 0.956	*p* = 0.832	*p* = 0.057
Egg mass, g/bird/day	9.41 ± 0.1	8.45 ± 0.3	9.70 ± 0.3	9.09 ± 0.4	*p* = 0.867	*p* = 0.903	*p* = 0.091
Feed intake, g	1,440.2^b^ ± 34.1	1,455.3^b^ ± 14.2	1,544.9^a^ ± 29.8	1,542.9^a^ ± 50.4	*p* = 0.002	*p* = 0.011	*p* = 0.009
FCR	0.68^b^ ± 0.0	0.78^a^ ± 0.0	0.71^ab^ ± 0.1	0.76^a^ ± 0.0	*p* = 0.195	*p* = 0.282	*P* = 0.041

**Notes.**

Means within a row with different superscripts differ significantly (*p* < 0.05). Data are presented as mean ± Standard Error of the Mean (SEM).

PH pistachio hull L Linear Q Quadratic C Cubic LW Live weight FCR Feed Conversion Ratio (feed/egg mass)

**Table 2:** Effect of pistachio hull addition on the quality characteristics of quail eggs.

**Traits**	**Control**	**PH 2%**	**PH 4%**	**PH 6%**	**L**	**Q**	**C**
Egg weight^*^, g	13.63 ± 1.3	14.22 + 1.1	13.92 + 1.4	13.94 + 0.9	*p* = 0.509	*p* = 0.325	*p* = 0.280
Shell weight, g	1.22 ± 0.1	1.17 ± 0.2	1.16 ± 0.1	1.19 ± 0.1	*p* = 0.248	*p* = 0.144	*p* = 0.276
Shell thickness, mm	0.25 ± 0.0	0.25 ± 0.0	0.25 ± 0.0	0.25 ± 0.0	*p* = 0.495	*p* = 0.573	*p* = 0.728
Shape index, %	77.90^b^+ 2.6	78.60^b^+ 3.2	80.44^a^+ 2.0	78.54^b^ ± 2.5	*p* = 0.093	*p* = 0.007	*p* = 0.002
Albumen index, %	15.05 + 2.4	14.78 ± 2.2	15.79 ± 2.2	14.33 ± 2.0	*p* = 0.535	*p* = 0.284	*p* = 0.078
Yellow index, %	47.75 ± 3.0	47.93 ± 3.3	48.33 ± 4.1	48.13 ± 3.7	*p* = 0.593	*p* = 0.830	*p* = 0.930
Haugh unit	98.43 ± 3.4	97.66 ± 3.2	98.95 ± 2.9	97.07 ± 2.7	*p* = 0.265	*p* = 0.331	*p* = 0.085
Yellow color	8.23^d^ ± 0.4	9.73^c^ ± 1.0	10.20^b^ ± 0.9	11.43^a^ ± 0.8	*p* = 0.000	*p* = 0.000	*p* = 0.000
Yellow weight, g	4.24 ± 0.4	4.47 ± 0.6	4.16 ± 0.6	4.21 ± 0.4	*p* = 0.322	*p* = 0.374	*p* = 0.076

**Notes.**

In each row, groups sharing the same letter (a, b) are not significantly different.

PH pistachio hull SEM standard error mean L Linear Q Quadratic C Cubic

* Egg weights given in this table only refer to the eggs weighed in 7th and 11th weeks.

### Performance characteristics of quails fed pistachio hull

Studentized residuals from the analysis of variance (ANOVA) models satisfied assumptions for most traits (Table S1; Fig. S1). Levene’s tests were non-significant for all performance characteristcs (all p ≈ 0.56−0.93), indicating homogeneous variances. Shapiro–Wilk tests supported residual normality for initial live weight (LW), daily egg performance, egg mass, average feed consumption, and feed conversion ratio (FCR) (all *p* > 0.05), whereas two traits exhibited departures: egg weight (*p* = 0.021) and final LW (*p* = 0.004). As a robustness check, Kruskal-Wallis tests were consistent with the ANOVA inference for egg weight (*H* = 24.07, *p* = 0.000), while for final LW the KW test was not significant (*H* = 7.336, *p* = 0.062), suggesting that the group effect on final LW should be interpreted cautiously. Overall conclusions for the trait set are otherwise unchanged.

The effects of dietary pistachio hull (PH) supplementation at 2%, 4%, and 6% inclusion levels on LW were monitored between 7–11 week laying period (Table 1). Initial LW did not differ significantly among treatments at the beginning of the experiment (*p* > 0.05). However, final LW exhibited a cubic trend (*p* = 0.05) across treatments. The PH 2% group (356.5 g) achieved the highest final LW, which was 6.8% greater than the control (333.9 g), though this difference was not statistically significant. Conversely, the PH 4% treatment (326.1 g) resulted in a 2.3% reduction in final LW compared to the control and differed significantly (*p* < 0.05) from both PH 2% and PH 6% (350.6 g), which showed a 5.0% increase over the control. These findings suggest that moderate-to-high PH inclusion (2–6%) may variably influence LW, with PH 4% potentially suppressing growth.

The inclusion of PH in the diet significantly influenced egg weight, with linear, quadratic, and cubic trends observed (*p* < 0.01). The highest egg weight was recorded in the PH 6% group (13.33 g), which was 6.1% greater than the control group (12.57 g), though this difference was not statistically significant. The PH 2% treatment (13.05 g) resulted in a 3.8% increase over the control, while PH 4% (12.91 g) showed a 2.7% increase. However, significant differences (*p* < 0.05) were detected between PH 4% and other treatments, as indicated by superscript letters, suggesting a complex dose–response relationship. The egg weights given here represent the egg weights taken every day between 7 and 11 weeks.

Egg yield exhibited variation across treatments, though no significant linear, quadratic, or cubic trends were observed (*p* > 0.05). The PH 4% group achieved the highest egg yield (76.4%), which was 2.0% higher than the control (74.9%) and significantly greater (*p* < 0.05) than the PH 2% group (66.0%), which showed an 11.9% reduction compared to the control. The PH 6% treatment (71.7%) yielded intermediate results, with a 4.3% decrease relative to the control. These findings indicate that moderate PH inclusion (4%) may enhance egg production, while higher doses (6%) or lower doses (2%) did not significantly differ from the control. Egg mass generally peaked during weeks 7-11, with the 4% PH group exhibiting the highest value (9.70 g). Although the control (9.41 g) and 6% PH (9.09 g) groups showed similar results, the 2% PH group had a lower egg mass (8.45 g). The overall average did not reach statistical significance (*P* > 0.05), and no linear or quadratic trends were observed, indicating that PH inclusion did not adversely affect egg mass.

Feed intake was significantly affected by PH level, with pronounced linear, quadratic, and cubic trends (*p* < 0.01, *p* = 0.05, and *p* < 0.01, respectively). Birds fed PH 4% (1544.9 g) and PH 6% (1542.9 g) consumed 7.3% and 7.1% more feed, respectively, compared to the control group (1440.2 g), with both treatments differing significantly (*p* < 0.05). The PH 2% group (1455.3 g) showed no significant difference from the control. These results suggest that higher PH inclusion levels (4–6%) stimulate feed consumption, potentially influencing metabolic or palatability factors.

FCR was significantly affected by PH supplementation, with a cubic trend (*p* = 0.05). The control group (0.68 kg) exhibited the lowest (most efficient) FCR, which was 14.7% better than the PH 2% group (0.78 kg) and 11.8% better than the PH 6% group (0.76 kg), both of which differed significantly (*p* < 0.05). The PH 4% treatment (0.71 kg) showed intermediate efficiency, with a 4.4% increase in FCR compared to the control, but no significant difference from other groups. This indicates that higher PH doses (2–6%) impair feed efficiency, with the most pronounced effects at 2% and 6% inclusion levels.

### Quality characteristics of quail eggs

Studentized residuals from the ANOVA model satisfied assumptions for most traits (Table S2; Fig. S2). Levene’s tests were non-significant for all variables (all *p* > 0.05), indicating homogeneous variances. Shapiro–Wilk tests showed residual normality for egg weight, egg weight index, yellow index, Haugh unit, yellow weight, and shell thickness (all *p* > 0.05), whereas shape index (*p* = 0.001), yellow color (*p* = 0.000), and shell weight (*p* = 0.000) exhibited departures. As a robustness check, Kruskal-Wallis tests led to the same inference as ANOVA: shape index and yellow color differed among groups (*H* = 19.52, *p* = 0.000; *H* = 87.05, *p* = 0.000), while shell weight remained non-significant (*H* = 9.26, *p* = 0.094).

Egg weight did not differ significantly among treatments (*p* > 0.05; no significant linear, quadratic, or cubic trends). The control group produced eggs weighing 13.63 g. Inclusion of 2% PH increased mean egg weight to 14.22 g, a 4.3% rise over control, while 4% and 6% PH yielded 13.92 (+2.1%) and 13.94 g (+2.3%), respectively (Table 2). Despite these numerical increases, all four treatment means share the same statistical grouping, confirming that PH supplementation up to 6% does not produce statistically detectable differences in egg weight.

Eggshell weight likewise showed no significant treatment effects (*p* > 0.05 across all trend tests). Average eggshell weight was 1.22 g in the control. Inclusion of 2%, 4%, and 6% PH yielded shell weights of 1.17 g (−4.1%), 1.16 g (−4.9%), and 1.19 g (−2.5%), respectively (Table 2). These slight reductions in shell mass across all PH levels were not statistically significant (*p* > 0.05), indicating that PH supplementation up to 6% does not adversely affect eggshell formation. Eggshell thickness remained uniform across all dietary treatments, averaging 0.25 mm in the control and identical values in the 2%, 4%, and 6% PH groups (Table 2). Numerical differences were negligible and statistical analysis confirmed no significant effect of PH inclusion on shell thickness (*p* > 0.05 for linear, quadratic, and cubic contrasts), indicating that adding up to 6% PH does not alter the deposition of shell minerals (as reflected by shell thickness).

The egg shape index was significantly affected by PH level (quadratic *p* < 0.01; cubic *p* < 0.01), though not in a simple linear fashion. Control eggs had a mean shape index of 77.90 while the 2% and 6% PH groups measured 78.60 (+0.9%) and 78.54 (+0.8%) respectively, differences that were not statistically distinct from control. In contrast, the 4% PH eggs averaged 80.44, a 3.3% increase and the only group to differ significantly from control (*p* < 0.01), suggesting optimal pistachio hull inclusion can modestly enhance egg symmetry or elongation. The significant quadratic and cubic trends reflect this peak at 4% and a return toward baseline at higher inclusion.

The albumen index showed only minor, non-significant fluctuations across treatments (*p* > 0.05). Control birds produced an albumen index of 15.05. Inclusion of 2% PH yielded a slightly lower value (14.78; −1.8%), while 4% PH gave the highest mean (15.79; +4.9%). At 6% PH, the index fell to 14.33 (−4.8%). Despite these numerical differences, and the grouping by letters (a, b) suggesting that the 4% and 6% PH means differ in pairwise comparison, ANOVA indicated no statistically significant effect of pistachio hull level on albumen index. These results imply that PH supplementation up to 6% does not meaningfully alter egg white consistency as measured by the albumen index.

Egg yolk color intensity, as quantified by the yellow index, remained stable across treatments, with mean values of 47.75 in the control, 47.93 at 2% PH (+0.4%), 48.33 at 4% PH (+1.2%), and 48.13 at 6% PH (+0.8%). Analysis of linear, quadratic, and cubic dose–response contrasts all returned *p* > 0.05, indicating no significant straight-line, U-shaped, or S-shaped relationship between PH level and yolk color. In other words, increasing PH up to 6% did not meaningfully shift yolk pigmentation. The Haugh unit, a standard measure of albumen quality, averaged 98.43 in control eggs, with slight variations of 97.66 at 2% PH (−0.8%), 98.95 at 4% PH (+0.5%), and 97.07 at 6% PH (−1.4%). None of the linear, quadratic, or cubic trends were statistically significant (*p* > 0.05), signifying that PH inclusion had no detectable effect on egg white viscosity or freshness as measured by the Haugh unit.

Yolk color score rose significantly with PH supplementation. Controls scored 8.23 ±0.4, increasing to 9.73 at 2% PH (+18.3%), 10.20 at 4% PH (+24.0%), and 11.43 at 6% PH (+38.9%). All three dose–response contrasts were highly significant (*p* < 0.01), indicating a complex relationship. A linear effect signifies that, on average, yolk color darkened progressively with each PH increment. A quadratic effect reveals curvature, suggesting that the rate of color change accelerated at intermediate levels before leveling off. A cubic effect uncovers even higher-order nuances, implying slight inflection points, perhaps a small plateau between 4% and 6% before final darkening. Together, these trends demonstrate that PH pigments or associated bioactives strongly enhance yolk coloration in a dose-dependent yet non-uniform manner. The proportion of yolk mass (yellow weight) averaged 4.24 g in controls and showed minimal changes at 2% PH (4.47 g; +5.4%), 4% PH (4.16 g; −1.9%), and 6% PH (4.21 g; −0.7%). None of the linear, quadratic, or cubic contrasts reached significance (*p* > 0.05), indicating that pistachio hull inclusion did not appreciably affect the proportion of yolk mass in the eggs.

## Discussions

### Nonlinear growth dynamics and feed efficiency in response to PH inclusion

The observed variation in live weight gain across PH inclusion levels may reflect a nonlinear response to dietary fiber. While no statistically significant overall effect was found, birds in the 4% PH group had numerically lower final body weights compared to those in the 2% and 6% groups. This intermediate response may be due to a temporary imbalance between fiber content and the birds’ digestive processing capacity, potentially hindering nutrient utilization. In contrast, higher PH inclusion (6%) may have stimulated compensatory adaptations, such as enhanced gut motility or microbial fermentation, resulting in more efficient energy extraction and improved weight gain.

This cubic dose–response trend may be attributed to distinct physiological adaptation thresholds in response to dietary fiber. At low inclusion levels, fiber can stimulate gastrointestinal motility and mucosal development, enhancing nutrient utilization. As fiber increases to moderate levels, it may begin to interfere with digestive efficiency by diluting energy density or disrupting nutrient absorption. However, at higher inclusion levels, compensatory mechanisms, such as increased microbial fermentation and adaptation of gut microbiota, may partially mitigate these negative effects. A cubic model, unlike linear or quadratic trends, captures this three-phase response (stimulation, inhibition, compensation), making it more appropriate for modeling complex biological adaptations to dietary fiber inclusion. The National Research Council (NRC) (1994) similarly emphasize the necessity of dosage optimization when incorporating high-fiber agricultural by-products into poultry diets. Comparative studies reinforce these findings. Kaya et al. (2013), Uddin et al. (2021), and Abu Hafsa et al. (2024) reported that moderate dietary fiber can enhance nutrient uptake and gut health, whereas excessive fiber impairs digestion. Ayobami (2024) further notes that fiber particle size influences gastrointestinal transit and microbial community structure, underlining the importance of feed formulation.

In other quail feed-additive trials, Abd El-Galil & Mahmoud (2015) found that ginger root powder (0.25−0.75 g/kg) significantly increased live weight by stimulating digestive enzymes. Djeddi (1999) reported weight gains at 5–15% vetch seed inclusion but weight decline at 20%, mirroring our intermediate-level decline. Vardar, Gökmen & Bahtiyarca (2020) observed no detrimental effects of anise seed (up to 22.5 g/kg) on live weight, though performance gains were also absent. Together, these studies highlight a common theme: plant–based feed additives exhibit non-linear dose responses in poultry, where both under- and over-inclusion can limit benefits. The findings contribute to the body of literature by demonstrating that PH, an abundant agro-industrial by-product, can safely improve final body weight in quails when inclusion levels are carefully calibrated.

### Egg production and metabolic adaptations under PH supplementation

The clear enhancement of egg weight with higher PH inclusion suggests that pistachio hulls favorably modulate energy metabolism and nutrient partitioning during egg formation. Fiber in PH may slow feed passage, increasing retention time and improving lipid and protein absorption, key constituents of egg yolk and albumen. Comparable findings in the literature support these mechanisms. Dotas, Symeon & Dublecz (2025) showed that high-fiber feeds optimized gut health and yolk development, while Wang, Cespedes-Acuña & Wei (2024) underscored the role of dietary energy sources in sustaining egg mass. In contrast, studies using other by-products have yielded mixed results: Djeddi (1999) observed no egg weight change with vetch seed at moderate inclusion levels, and Mohammadabadi & Zarei (2018) and Vardar, Gökmen & Bahtiyarca (2020) reported similarly neutral effects with pennyroyal and anise seed, respectively.

However, potent bioactive additives, such as garlic powder (Olayinka et al., 2022), red pepper pigment (Li et al., 2012), and ginger root (Abd El-Galil & Mahmoud, 2015), produced marked egg weight gains through enhanced metabolic pathways, allicin and sulfur compounds in garlic, carotenoids in red pepper, and digestive enzyme stimulation by ginger. The significant egg-weight increases at both low and high PH levels in our study align more closely with these bioactive interventions, suggesting that PH contains bioactive constituents (*e.g.*, phenolics, tannins) that may similarly enhance metabolic efficiency. The nonlinear (cubic) dose–response observed underscores the complexity of fiber-based additives: moderate levels can optimize gut function and egg synthesis, but excessive fiber may overwhelm digestive capacity or alter nutrient digestibility. Thus, precise formulation of PH inclusion is critical.

Although the differences in egg-laying performance were not statistically significant, the modest gain observed in the 4% PH group suggests a potential benefit of moderate pistachio hull inclusion. At this level, PH may provide an optimal blend of fermentable fiber and energy, supporting sustained egg output without overburdening digestive processes.

Previous work underscores the importance of both additive type and dosage. Djeddi (1999) found that vetch seed inclusions up to 15% had no adverse effect on quail egg production, while a 20% inclusion markedly reduced productivity, mirroring our finding that higher PH levels may risk digestive inefficiencies. In contrast, Abd El-Galil & Mahmoud (2015) reported significant boosts in egg production with ginger root supplementation (0.25–0.75 g kg^−^^1^), attributing the effect to ginger’s bioactive compounds (gingerol, shogaol) that enhance metabolic activity.

Not all plant-derived additives yield measurable improvements. Li et al. (2012) observed no egg-production change with red pepper pigment (1.2–9.6 mg kg^−^^1^), and Vardar, Gökmen & Bahtiyarca (2020) similarly reported neutral outcomes for anise seed (22.5 g kg^−^^1^). These mixed results highlight that fiber source, particle size, and bioactive content vary widely in their physiological effects. Taken together, our data and the literature emphasize that moderate PH inclusion (around 4%) may support egg production by balancing additional fermentable substrate and energy supply. Exceeding this threshold, however, risks digestive overload without further performance gains.

### Feed intake and efficiency

The relationship between dietary additives and feed utilization patterns in poultry exhibits considerable complexity, as evidenced by divergent findings across studies. For instance, Djeddi (1999) demonstrated that incremental inclusion of common vetch (*Vicia sativa* L.) at 5%, 10%, and 15% in quail diets significantly elevated feed intake relative to controls, whereas a 20% inclusion level paradoxically suppressed consumption. This nonlinear response underscores the delicate balance between beneficial nutrient contributions and potential anti-nutritional effects at higher doses. Similarly, Bhawa, Moreki & Manyeula (2025) observed that tomato waste supplementation at graded levels enhanced overall performance metrics in Japanese quails without significantly altering feed intake, suggesting that nutrient bioavailability, rather than intake volume, may drive productivity in such formulations. In contrast, Saraçoğlu et al. (2025) documented pronounced improvements in both feed intake and FCR following the incorporation of capsaicin-based additives, likely attributable to the compound’s stimulatory effects on digestive enzyme activity and appetite regulation. Conversely, Mohamed et al. (2025) reported no statistically significant shifts in feed consumption with microalgae supplementation, highlighting the context-dependent nature of additive efficacy.

These disparities in feed intake responses likely arise from intrinsic structural and metabolic properties of the additives themselves. For example, fibrous components may modulate gut transit time, while bioactive compounds (*e.g.*, capsaicin, polyphenols) could influence metabolic pathways or microbial symbiosis. Such mechanisms are not exclusive to quails; analogous trends have been observed across poultry species. Ghorbani et al. (2025) illustrated that methionine supplementation in quail diets mitigated aflatoxicosis-induced dysbiosis, indirectly stabilizing feed intake through enhanced gut microbiota diversity and immune function. Similarly, Sultan et al. (2024) attributed improved growth performance and intestinal integrity in ginger-supplemented quails to the enzymatic activity of zingibain, which optimizes nutrient hydrolysis and absorption efficiency. Furthermore, Nasir et al. (2024) identified oyster mushroom waste as a dual-purpose additive, enhancing both feed consumption and disease resistance, a phenomenon potentially linked to *β*-glucans modulating immune-metabolic crosstalk.

The variability in feed conversion efficiency across studies further underscores the multifactorial nature of dietary interventions. Chen et al. (2025) reported neutral effects of pomegranate peel inclusion on FCR in poultry, positing that its high lignin content may offset potential benefits by reducing nutrient digestibility. Oketch et al. (2025) similarly found no significant FCR alterations with multiprotease supplementation in laying hens, suggesting that enzyme efficacy may depend on substrate specificity or synergistic interactions with other dietary components. Conversely, Sultan et al. (2024) achieved optimal FCR at 0.50 g/kg ginger root inclusion in quails, emphasizing the role of bioactive phytochemicals in streamlining energy partitioning. Such discrepancies likely stem from differences in additive composition (*e.g.*, fiber type, phenolic profiles), digestive adaptability of the host species, and interactions with basal diet matrices. Recent investigations into novel additives, including black soldier fly larvae meal (Pabillore et al., 2025) and ahiflower seed (Ogory, Cumberford & Adewole, 2025), further illustrate the intricate interplay between nutrient density, anti-nutritional factors, and metabolic plasticity in shaping feed efficiency outcomes.

Collectively, these findings underscore the necessity of a holistic, additive-specific approach to feed formulation. Structural attributes (*e.g.*, particle size, fiber solubility) and metabolic modifiers (*e.g.*, enzymes, secondary metabolites) jointly dictate the net impact on intake and efficiency, necessitating rigorous characterization of novel ingredients to optimize their inclusion in poultry diets. In our study, although PH inclusion led to a moderate increase in feed intake at certain levels, this was not consistently reflected in proportional improvements in egg weight or overall egg quality. For instance, while the 2% PH group showed slightly better feed conversion and egg mass compared to control, higher inclusion levels (4% and 6%) did not yield additional benefits, despite elevated intake. This suggests that increased feed intake alone does not guarantee enhanced productive output, particularly when the additive contains fibrous or polyphenol-rich components that may interfere with nutrient utilization. Therefore, the balance between intake volume and nutrient efficiency is critical, and our findings reinforce the importance of evaluating both feed utilization and output quality when assessing novel feed additives.

In light of our findings, it is important to interpret feed intake not in isolation, but in conjunction with performance outcomes such as egg mass and feed efficiency. Although PH inclusion led to a moderate increase in feed intake at certain levels, this was not consistently reflected in proportional improvements in FCR or egg mass. Egg mass is a key parameter that simultaneously reflects both egg production and egg size in quails. While PH did not negatively affect egg mass, it appears that the birds required some time to adapt to the increased fiber and phenolic content. This aligns with previous reports suggesting that high-fiber by-products may initially impair digestibility due to anti-nutritional compounds (Leeson & Summers, 2005). Nevertheless, our findings suggest that when appropriately dosed, PH inclusion can support performance without compromising productivity. Similarly, Salajegheh et al. (2012) found that dried tomato pulp enhanced egg mass, but emphasized the importance of precise dosage. Kim & Kang (2022) also showed that the nutritional balance of the entire diet plays a crucial role in determining egg productivity. Thus, while increased feed intake was observed at some PH levels, this alone did not drive improvements in egg parameters, underscoring the need to evaluate both nutrient efficiency and output quality when assessing the efficacy of novel feed additives.

### Egg mass

Egg mass is a key parameter that simultaneously reflects both egg production and egg size in quails. Addition of PH to the diet did not negatively affect egg mass, but it took some time for the quails to adapt to the PH doses. In the literature, it has been reported that certain by-products, due to their high fiber and phenolic content, may initially impair digestibility (Leeson & Summers, 2005). However, it appears that the inclusion of PH at appropriate levels may support performance. Similarly, Salajegheh et al. (2012) reported that dried tomato pulp could enhance egg mass, but emphasized the critical importance of dosage. Additionally, Kim & Kang (2022) revealed that the overall balance of the diet plays a significant role in influencing egg productivity.

### Effects of pistachio hull inclusion on egg physical and internal quality traits

The 4% PH group demonstrated the closest approximation to the ideal egg shape index (80.44), 3.3% higher than the control, suggesting enhanced mechanical resilience during handling and transport. This aligns with Abd El-Galil & Mahmoud (2015), who linked ginger root supplementation to improved shape indices in quail eggs, likely through bioactive compounds optimizing shell membrane elasticity. Although no significant differences were detected in albumen index, yolk index, or Haugh unit (*p* > 0.05), the 4% PH group exhibited a 4.9% higher albumen index and the highest Haugh unit value (98.95), indicative of superior protein stability and moisture retention in the albumen. These findings parallel (Olayinka et al., 2022), who observed a 44.2% increase in albumen index with garlic powder, and Mohammadabadi & Zarei (2018), who attributed antioxidant-rich pennyroyal to improved Haugh units. However, the lack of universal agreement, such as Djeddi’s (1999) reported declines in albumen quality at high vetch inclusion (20%) and Abd El-Galil & Mahmoud’s (2015) inverse relationship between ginger concentration and albumen/yolk indices, highlights the delicate balance required in additive dosing. These discrepancies underscore the importance of additive-specific mechanisms, where PH’s fiber matrix or phenolic content may stabilize albumen structure without overwhelming digestive processes at moderate inclusion levels.

### Pigmentation dynamics and shell characteristics: additive-specific interactions

The linear, quadratic, and cubic increases in yolk color score with PH supplementation (*p* < 0.01), peaking at 6% PH (38.9% above control), strongly implicate carotenoids or other pigments in pistachio hulls as drivers of enhanced yolk pigmentation. The positive effect of PH on yolk pigmentation observed in our study may be attributed to the high content of natural pigments and antioxidants. These results are consistent with studies using other plant-derived feed components rich in similar bioactives. For example, a study by Flores et al. (2022) showed that alternative plant feed ingredients did not negatively impact laying performance while potentially influencing yolk color outcomes, depending on the inclusion level. This aligns with Dosoky et al. (2021), who attributed yolk color improvements to onion and cinnamon pigments, and Li et al. (2012), who documented dose-dependent carotenoid effects from red pepper. Similarly, Mohammadabadi & Zarei (2018) linked *Mentha pulegium*’s carotenoid content to yolk pigmentation, reinforcing the role of plant-derived pigments in modulating visual egg quality. Despite these pigmentation benefits, yolk weight remained statistically unchanged across groups, mirroring (Dosoky et al., 2021) but contrasting with studies showing additive-driven yolk mass shifts (*e.g.*, garlic in Olayinka et al., 2022).

The absence of significant differences in egg weight, shell weight, and shell thickness across PH groups mirrors findings for ginger root (Abd El-Galil & Mahmoud, 2015) and *Capsicum frutescens* (Li et al., 2012), suggesting that PH does not directly influence calcification pathways. However, Mohammadabadi & Zarei (2018) reported increased shell thickness with *Mentha pulegium*, likely due to its mineral-rich profile or antioxidant-mediated calcium absorption. These contrasts emphasize that shell quality responses are additive-specific, hinging on interactions between dietary components (*e.g.*, fiber, minerals) and physiological processes such as calcium metabolism or oxidative stress regulation.

### Implications and future directions

The findings of this study suggest that PH, when included at appropriate levels, offer a sustainable and functional feed additive option for quail production. Their high pigment and fiber content contributed to enhancements in yolk coloration and egg shape index, while not adversely affecting eggshell quality or internal egg components.

For practical applications, a 4% PH inclusion is suitable for optimizing egg yield and shape index, while a 6% inclusion may benefit producers seeking improved yolk pigmentation, provided the minor decrease in feed conversion efficiency is economically tolerable.

Further research should aim to elucidate the mechanisms underlying these effects. Advanced biochemical techniques, such as high-performance liquid chromatography or metabolomic profiling, may identify bioactive constituents (*e.g.*, polyphenols, flavonoids, carotenoids) responsible for improvements in pigmentation and performance.

Long-term studies are also needed to assess PH’s influence on reproductive performance, health, and longevity. Additionally, economic evaluations should consider the trade-off between improved egg quality and slightly reduced feed efficiency. Finally, expanding the application of PH to other poultry species (*e.g.*, chickens, ducks) and different production systems (*e.g.*, organic, free-range) could broaden its utility. The potential to combine PH with enzymes (*e.g.*, phytase) or probiotics may help reduce anti-nutritional effects and enhance nutrient utilization. These strategies align with the broader goals of circular economy and reducing the environmental footprint of poultry production.

## Conclusions

This study demonstrated that PH, when included at 2–6% in the diets of laying quails, influence productivity and egg quality in a nonlinear, dose-dependent manner. The 6% PH level enhanced egg weight and yolk pigmentation, while 4% PH improved egg yield and shape index. However, higher inclusion levels slightly reduced feed efficiency. PH did not adversely affect eggshell quality or internal egg parameters, indicating its safe use as a sustainable feed additive. For optimal outcomes, a 4–6% inclusion level is recommended depending on production goals. Future studies should further explore the bioactive content, long-term effects, and economic feasibility of PH use in poultry diets.

This study demonstrated that PH, when included at 2–6% in the diets of laying quails, affect productivity and egg quality in a nonlinear, dose-dependent manner. The 6% PH level enhanced egg weight and yolk pigmentation, while 4% PH improved egg yield and shape index. It is worth noting that the term “egg weight” was evaluated in two contexts: average weekly egg weights during the laying period, and weights of selected eggs used for quality assessment at the 7th and 11th weeks. Higher inclusion levels slightly reduced feed efficiency. PH did not adversely affect eggshell quality or internal egg parameters, indicating its safe use as a sustainable feed additive. For optimal outcomes, a 4–6% inclusion level is recommended depending on production goals. Future studies should further explore the bioactive content, long-term effects, and economic feasibility of PH use in poultry diets.

The findings obtained in this study are not applicable to humans, but can be applied to experimental animals such as broiler chickens, laying hens and turkeys. All study data is accessible and the location of access is at my discretion.

##  Supplemental Information

10.7717/peerj.20204/supp-1 Supplemental Information 1 Raw data

10.7717/peerj.20204/supp-2 Supplemental Information 2 Residual Diagnostics for ANOVA Normality check of the dataset used in statistical evaluations.

10.7717/peerj.20204/supp-3 Supplemental Information 3 ARRIVE Checklist

## Additional Information and Declarations

### Data Availability

The following information was supplied regarding data availability: The raw measurements are available in the Supplemental File.
