# Supplementary material for: Utilization of soft pistachio hulls in Japanese quail diets for enhanced egg quality and yolk pigmentation
Source: PeerJ. 2025 Nov 21;13:e20204. doi: 10.7717/peerj.20204 (PMC12642913; doi:10.7717/peerj.20204)
Supplement: Supplemental Information 2 — Normality check of the dataset used in statistical evaluations. [file peerj-13-20204-s002.docx]

# Residual Diagnostics for ANOVA

For each trait, we fitted a one-way ANOVA model with Group (Control, PH2%, PH4%, PH6%) as the factor. We evaluated the model residuals as required by ANOVA assumptions: normality using the Shapiro-Wilk test on studentized residuals (with Q-Q plots), and homogeneity of variances using Levene’s test (median-centered) on the raw response across groups. Kruskal-Wallis tests were additionally reported as a robustness check for endpoints with significant departures from residual normality. Percentages are two-sided p-values; pass/fail thresholds are 0.05.

Table S1. Shapiro–Wilk and Levene’s tests on ANOVA studentized residuals for each trait shown in Table 1; ANOVA and Kruskal-Wallis p-values for Group.

| Sheet | N | k  Groups | Shapiro–Wilk W | **Shapiro p** | Levene stat | **Levene p** | ANOVA p | Kruskal H | **Kruskal p** |
| --- | --- | --- | --- | --- | --- | --- | --- | --- | --- |
| ILW | 108 | 4 | 0.977 | **0.057** | 0.657 | **0.581** | 0.315 | 3.95 | **0.267** |
| FLW | 96 | 4 | 0.958 | **0.004** | 0.686 | **0.563** | 0.026 | 7.336 | **0.062** |
| EW | 581 | 4 | 0.994 | **0.021** | 0.484 | **0.693** | 0.000 | 24.07 | **0.000** |
| DEP | 12 | 4 | 0.934 | **0.427** | 0.144 | **0.931** | 0.057 | 6.111 | **0.106** |
| EM | 12 | 4 | 0.947 | **0.596** | 0.211 | **0.886** | 0.090 | 5.821 | **0.121** |
| AFC | 12 | 4 | 0.979 | **0.980** | 0.498 | **0.694** | 0.009 | 8.401 | **0.038** |
| FCR | 12 | 4 | 0.951 | **0.655** | 0.541 | **0.667** | 0.044 | 6.795 | **0.079** |

IWB: Initial Live Weight; FLW: Final Live Weight; EW: Egg Weight; DEP: Daily Egg Performance; EM: Egg Mass; AFC: Awerage Feed Consumption: FCR: Feed Conversion Ratio


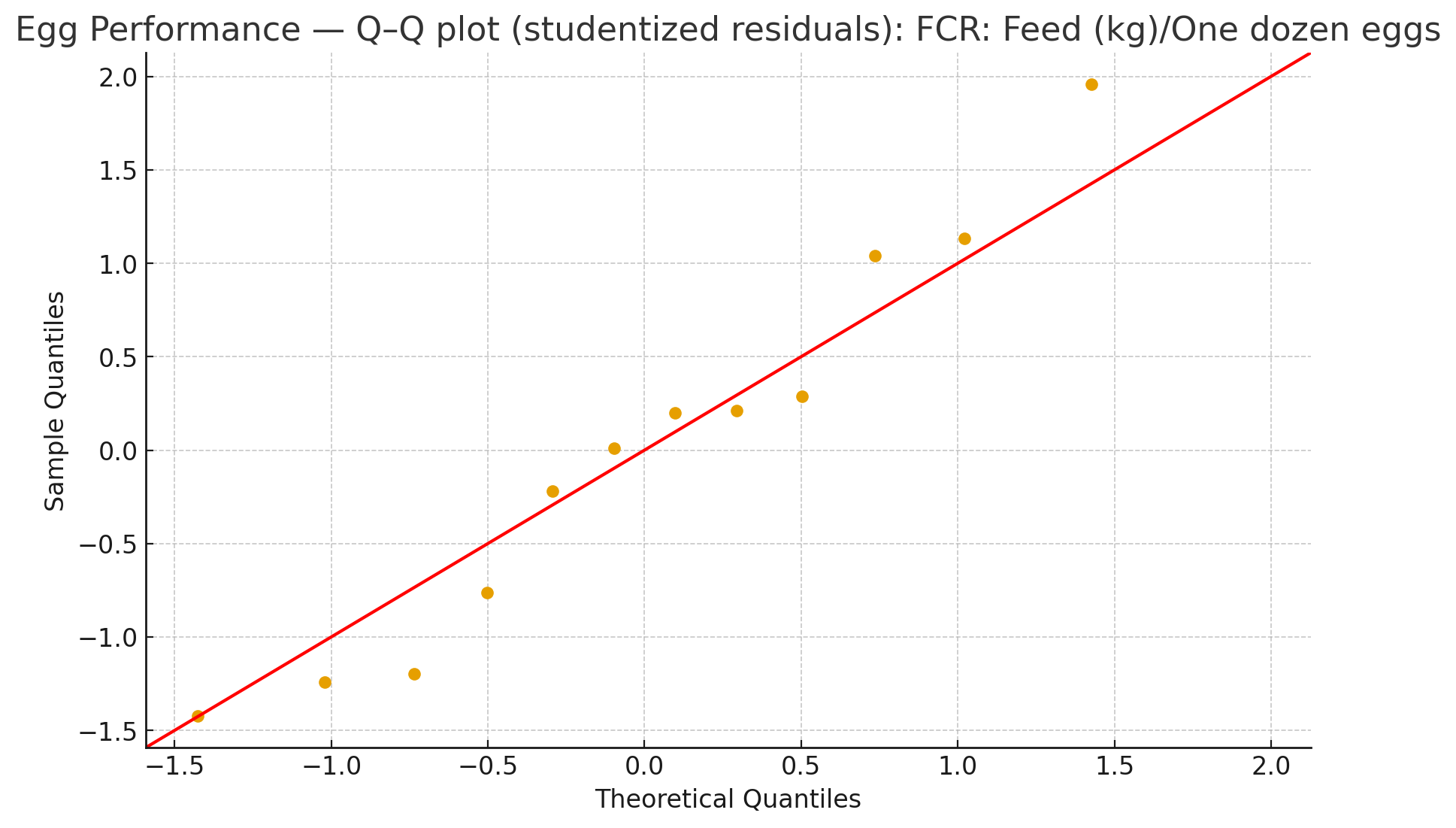

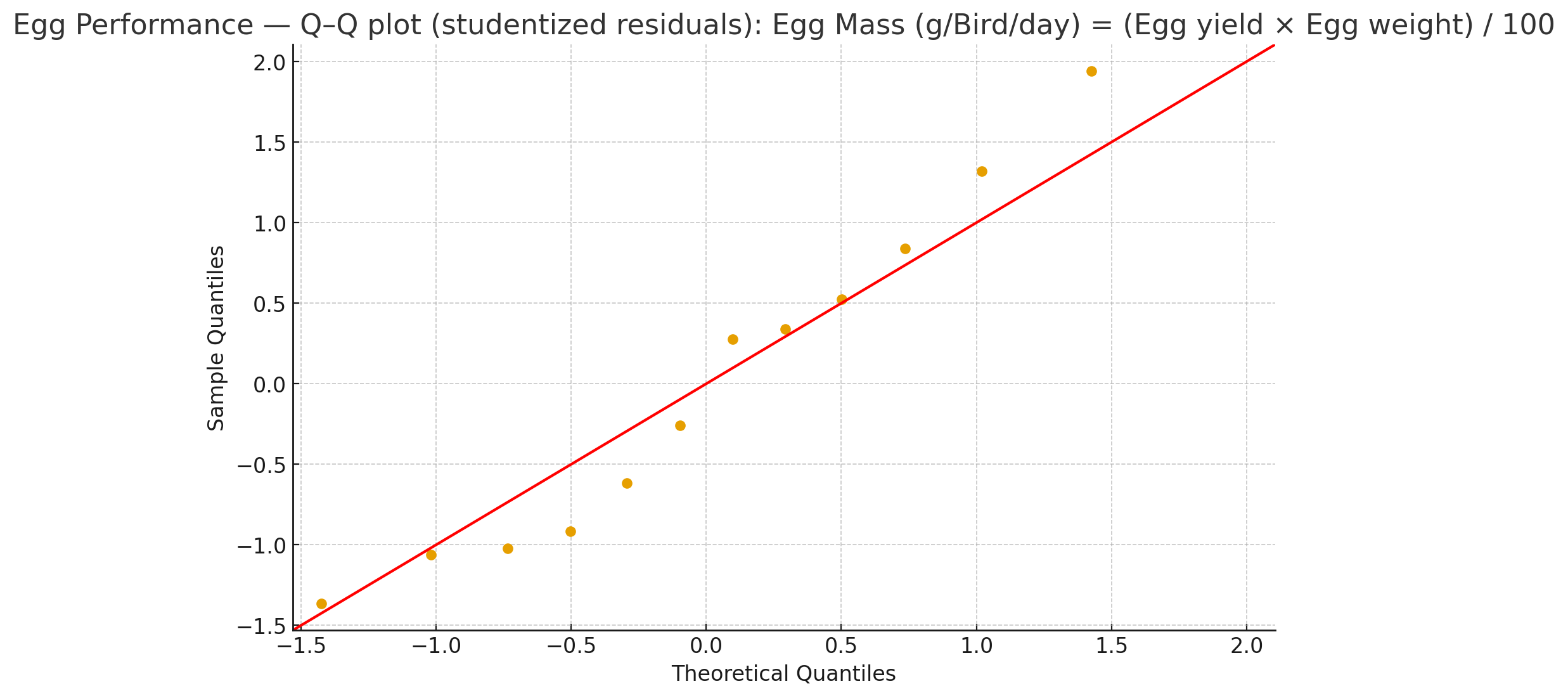

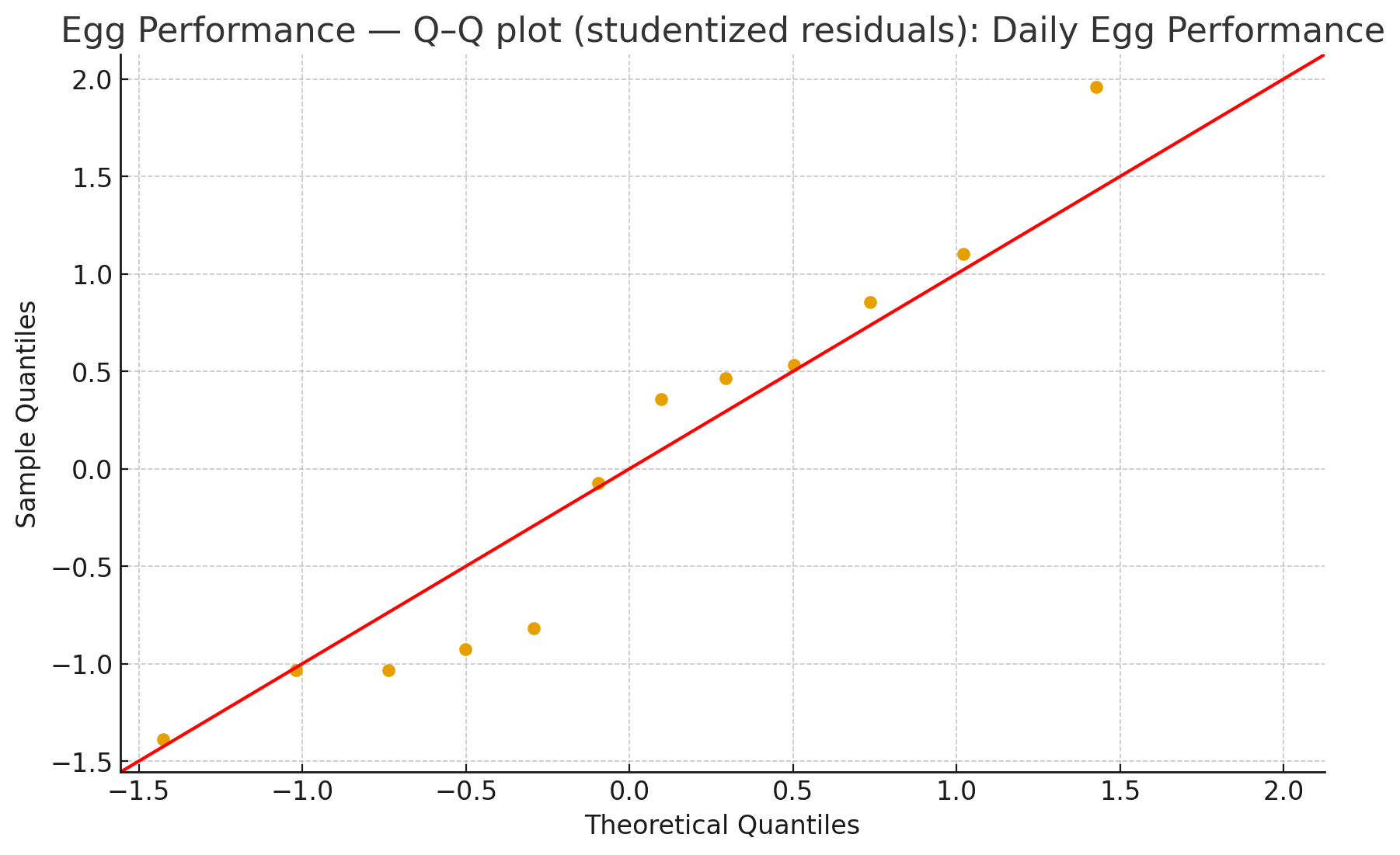

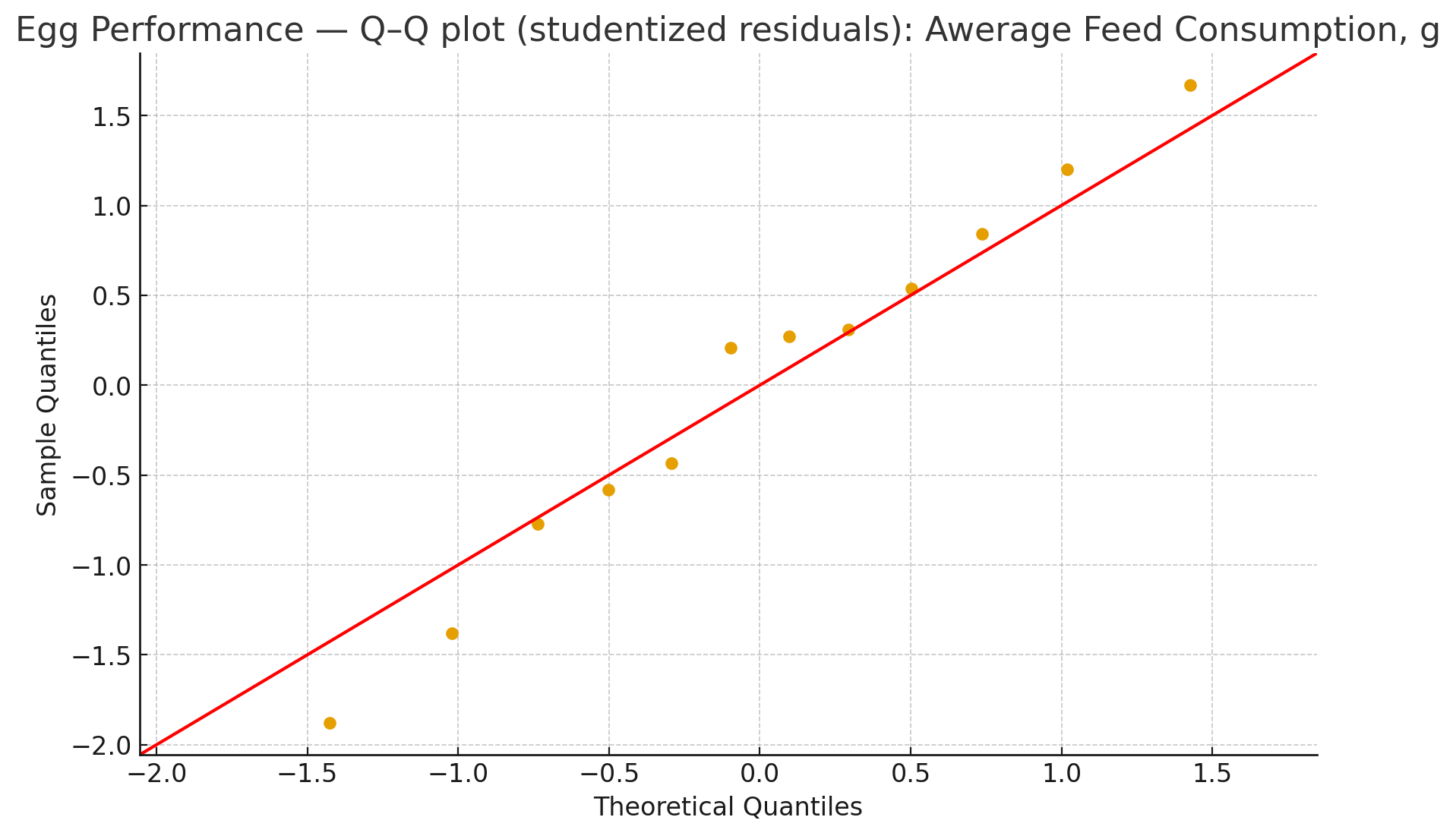

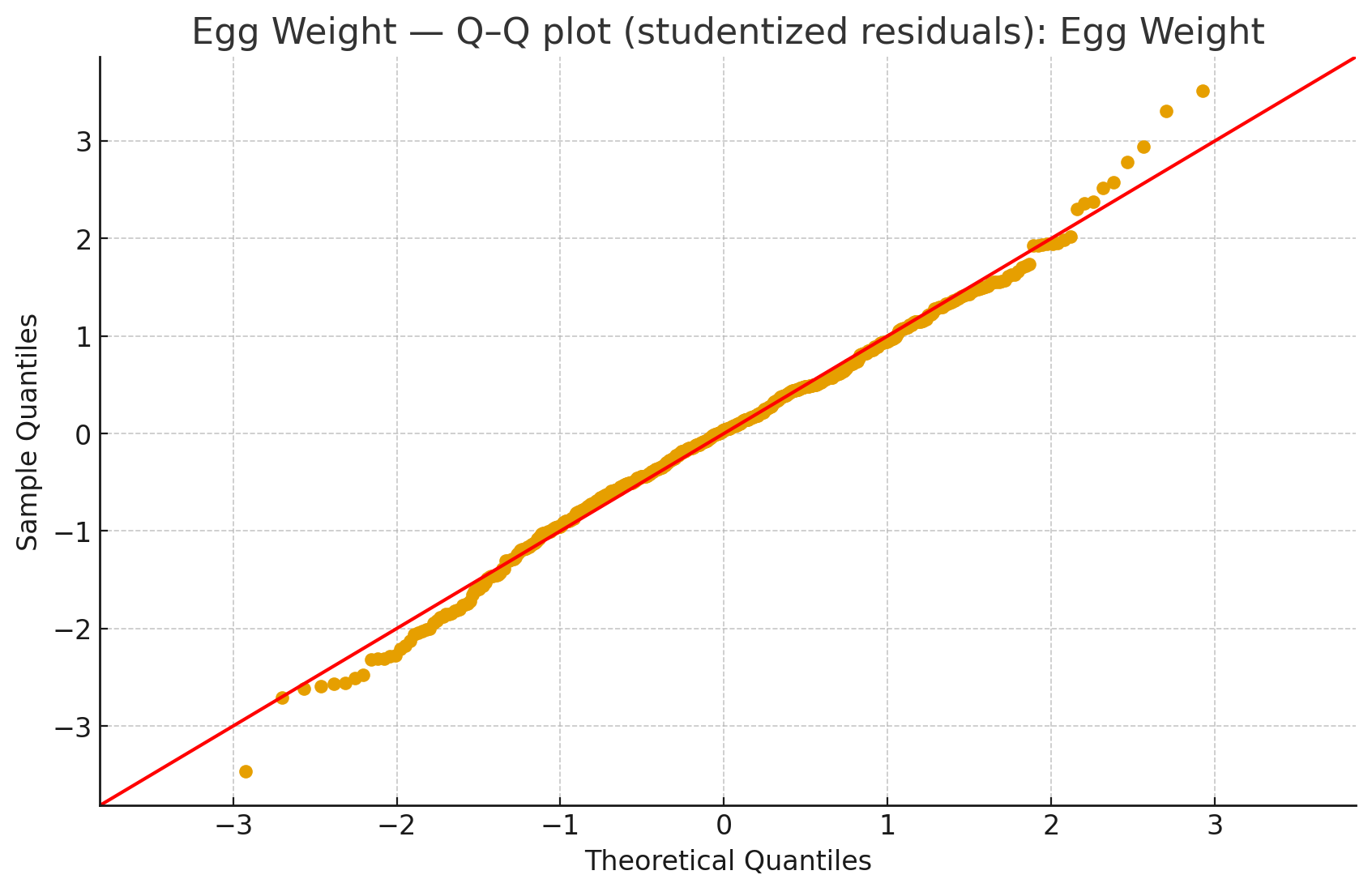


Fig. S1. Q–Q plots of studentized residuals for each trait shown in Table 1.

Table S2. Shapiro–Wilk and Levene’s tests on ANOVA studentized residuals for each trait shown in Table 2; ANOVA and Kruskal-Wallis p-values for Group.

| Response | N | k Groups | Shapiro-Wilk W | **Shapiro p** | Levene stat | Levene p | **ANOVA p** | Kruskal H | **Kruskal p** |
| --- | --- | --- | --- | --- | --- | --- | --- | --- | --- |
| EG | 120 | 4 | 0.985 | **0.192** | 1.930 | 0.130 | **0.287** | 3.920 | **0.270** |
| SI | 120 | 4 | 0.959 | **0.001** | 0.320 | 0.810 | **0.002** | 16.840 | **0.000** |
| EWI | 120 | 4 | 0.995 | **0.932** | 0.411 | 0.745 | **0.079** | 8.154 | **0.043** |
| YI | 120 | 4 | 0.987 | **0.281** | 1.351 | 0.261 | **0.930** | 0.649 | **0.885** |
| HU | 120 | 4 | 0.999 | **0.532** | 0.721 | 0.541 | **0.085** | 7.222 | **0.065** |
| YC | 120 | 4 | 0.893 | **0.000** | 2.109 | 0.103 | **0.000** | 87.010 | **0.000** |
| YW | 120 | 4 | 0.986 | **0.253** | 1.584 | 0.197 | **0.076** | 4.319 | **0.229** |
| SW | 120 | 4 | 0.795 | **0.000** | 1.165 | 0.326 | **0.276** | 6.394 | **0.094** |
| ST | 120 | 4 | 0.990 | **0.563** | 1.786 | 0.153 | **0.660** | 1.684 | **0.641** |

EG: Egg Weight; SI: Shape Index; EWI: Egg Weight Index; YI: Yellow Index; HU: Yellow Index; YC: Yellow Index; YW: Yellow Weight; SW: Shell Weight; ST: Shell Thickness

Q–Q plots of studentized residuals for each trait are as follows:


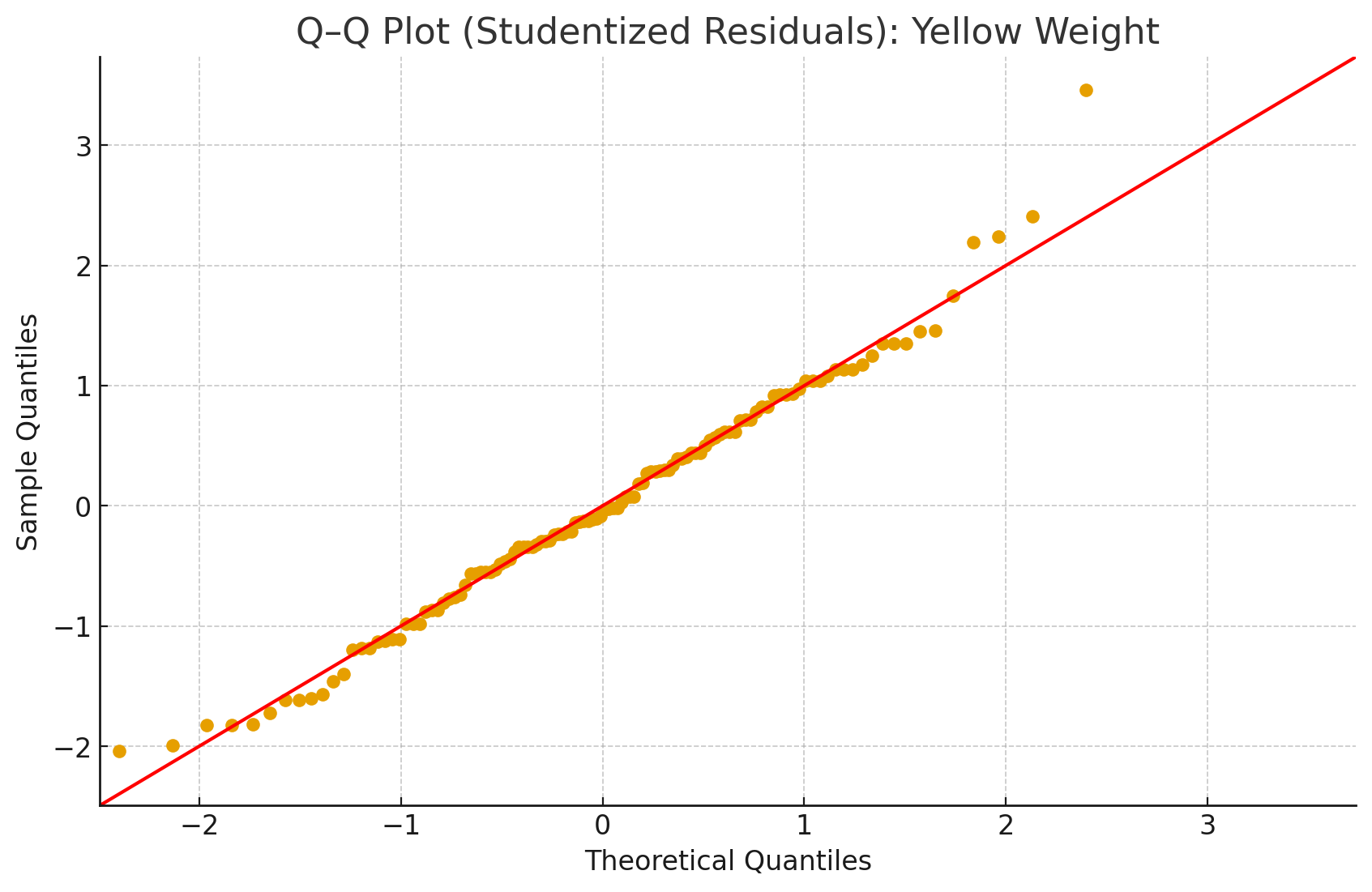

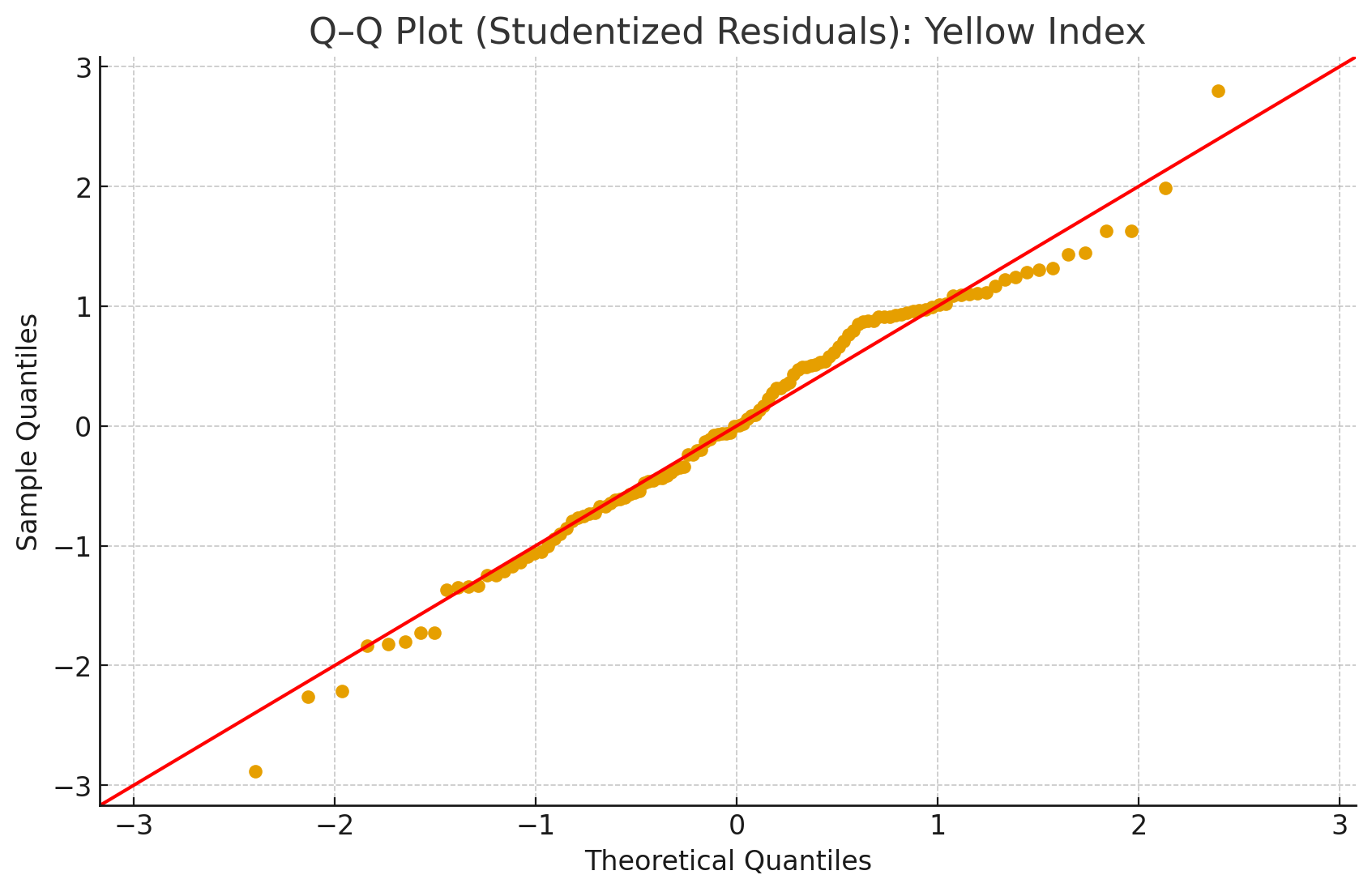

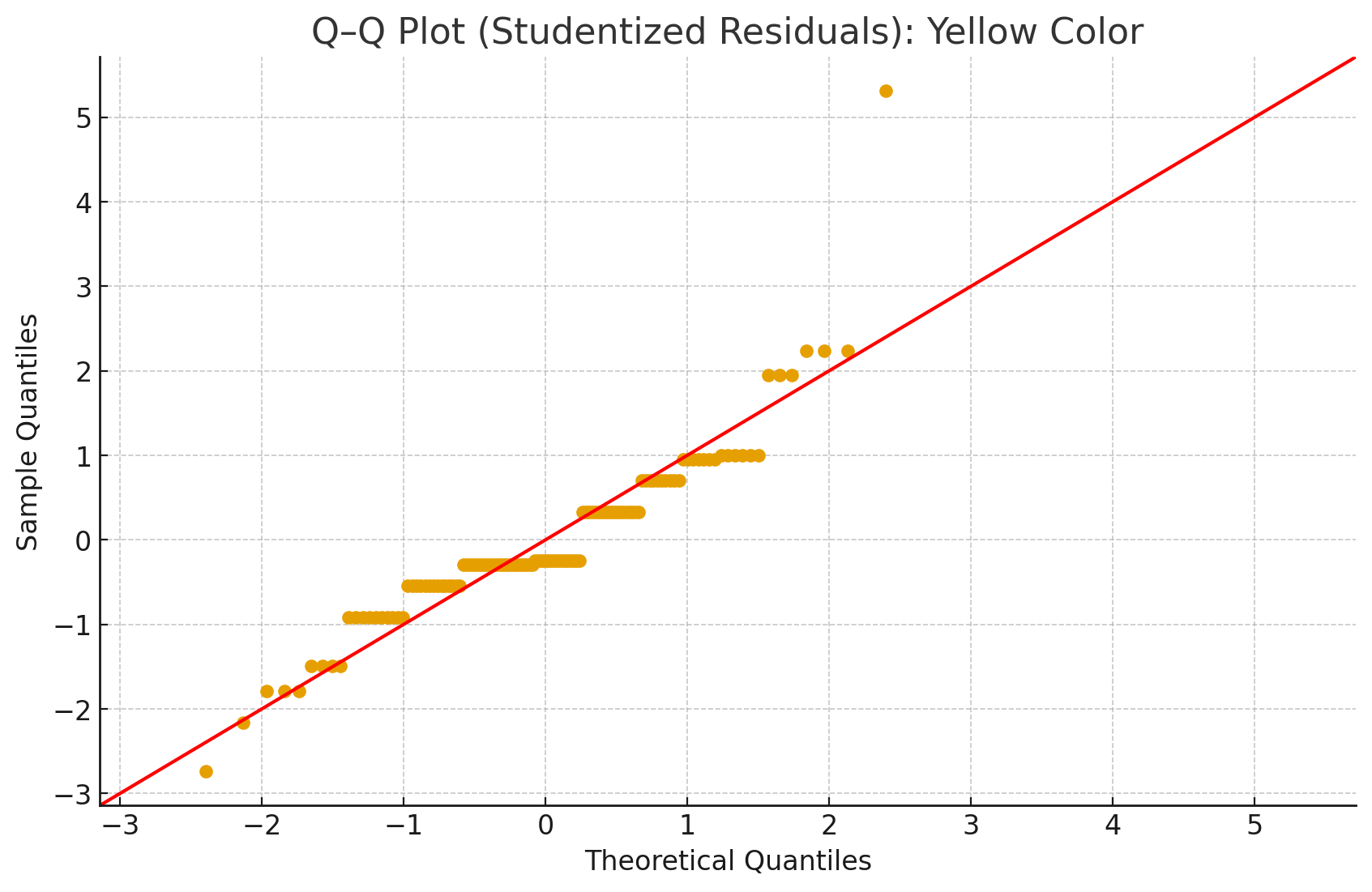

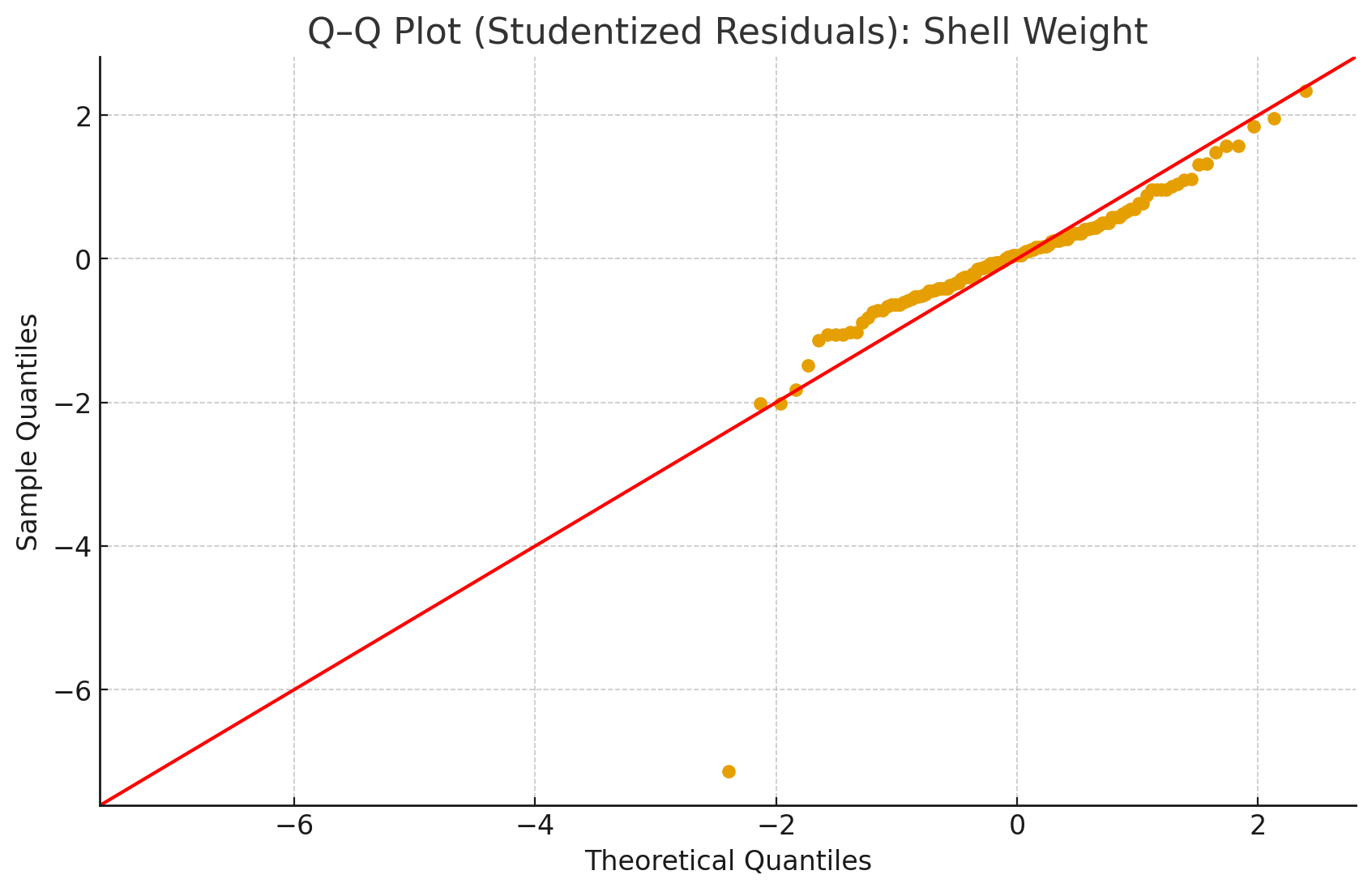

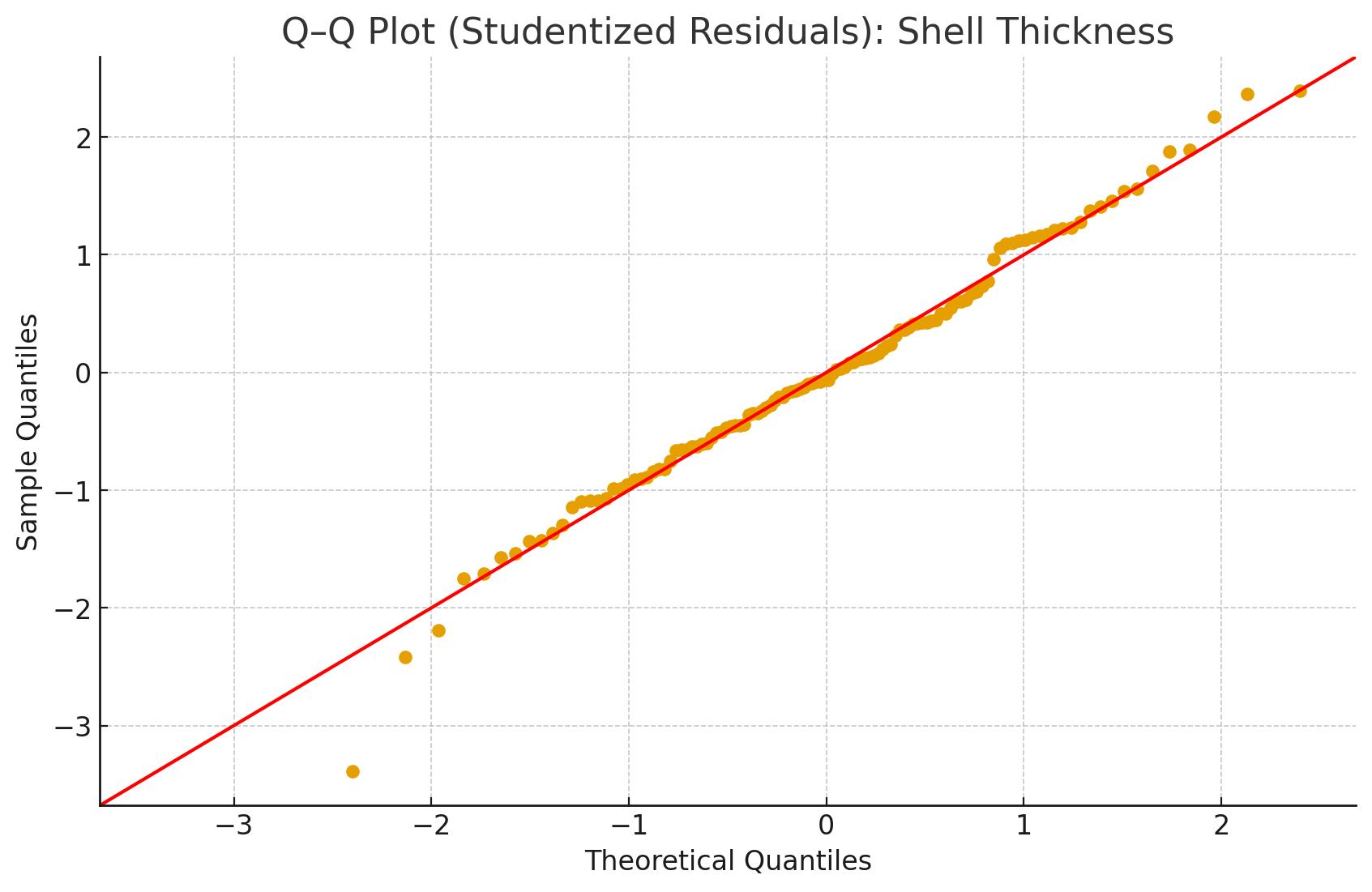

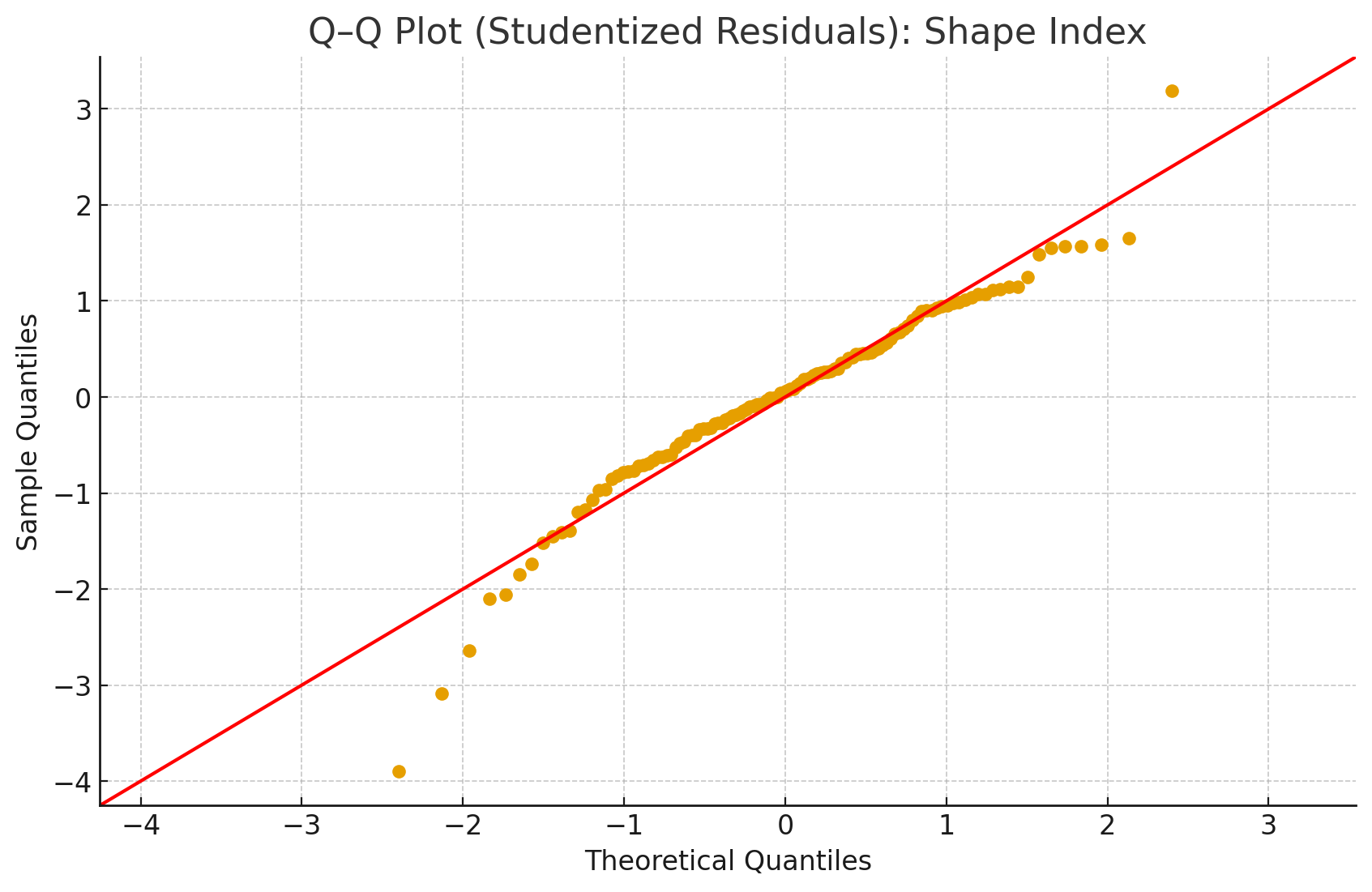

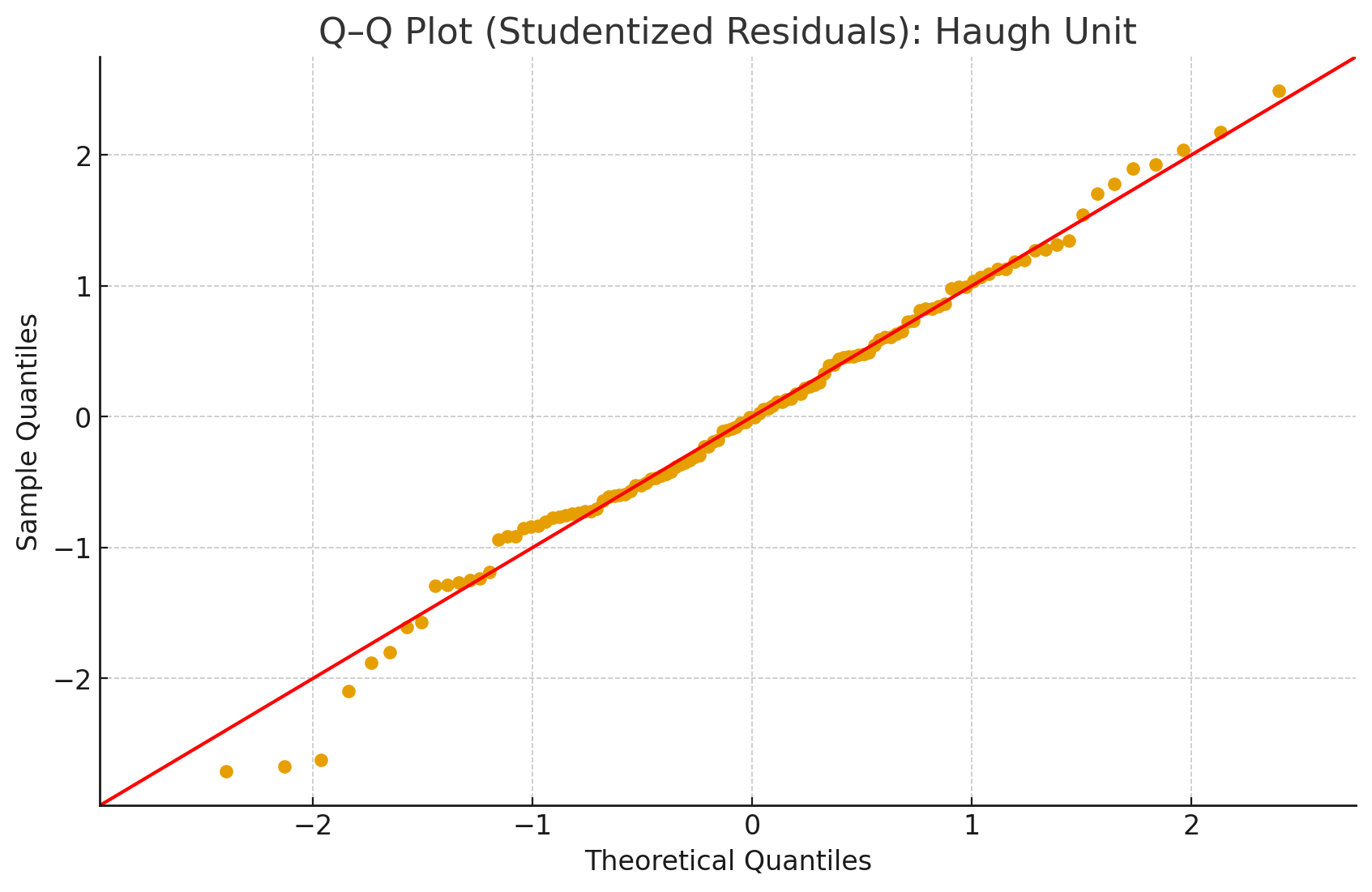

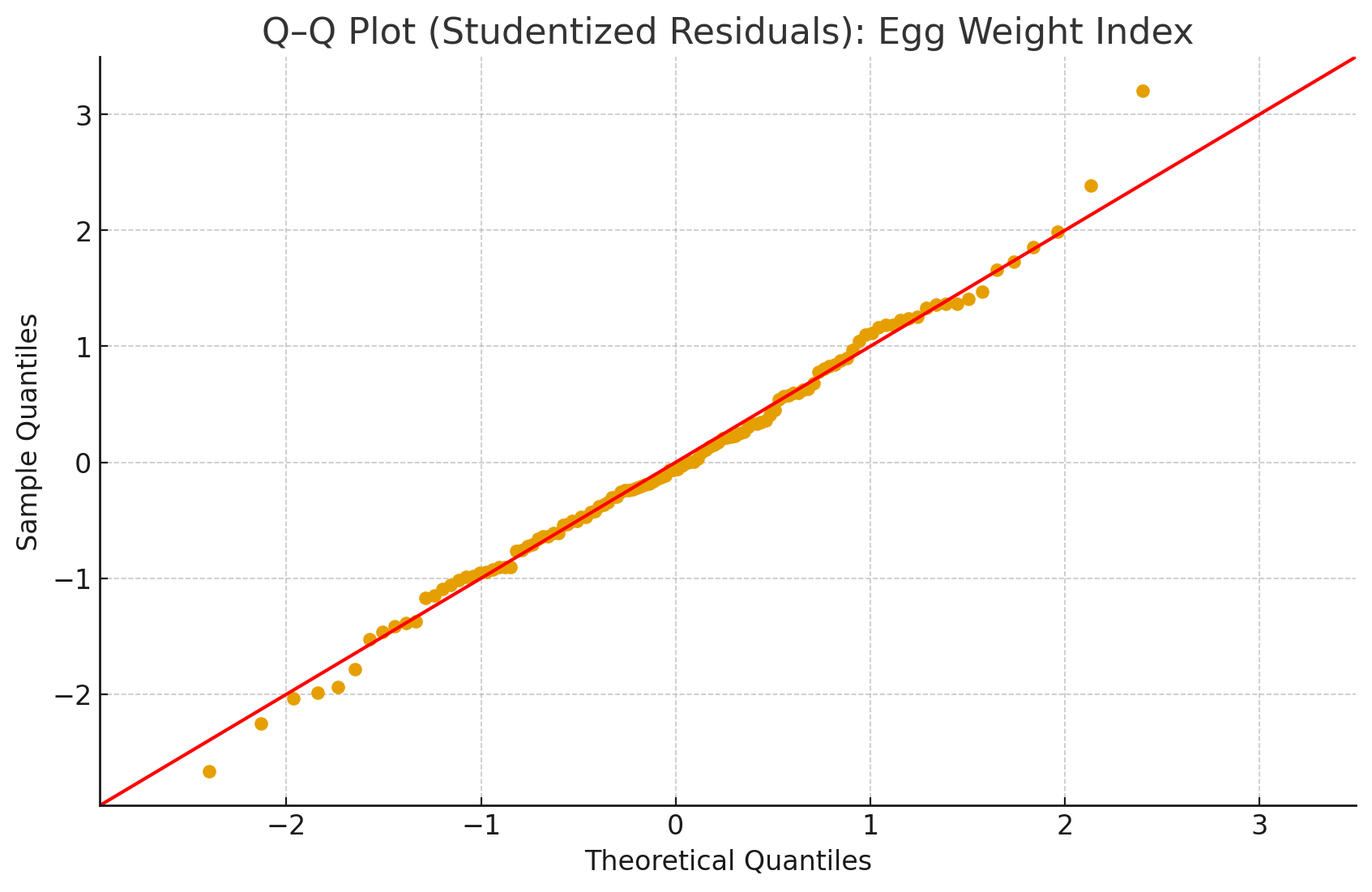

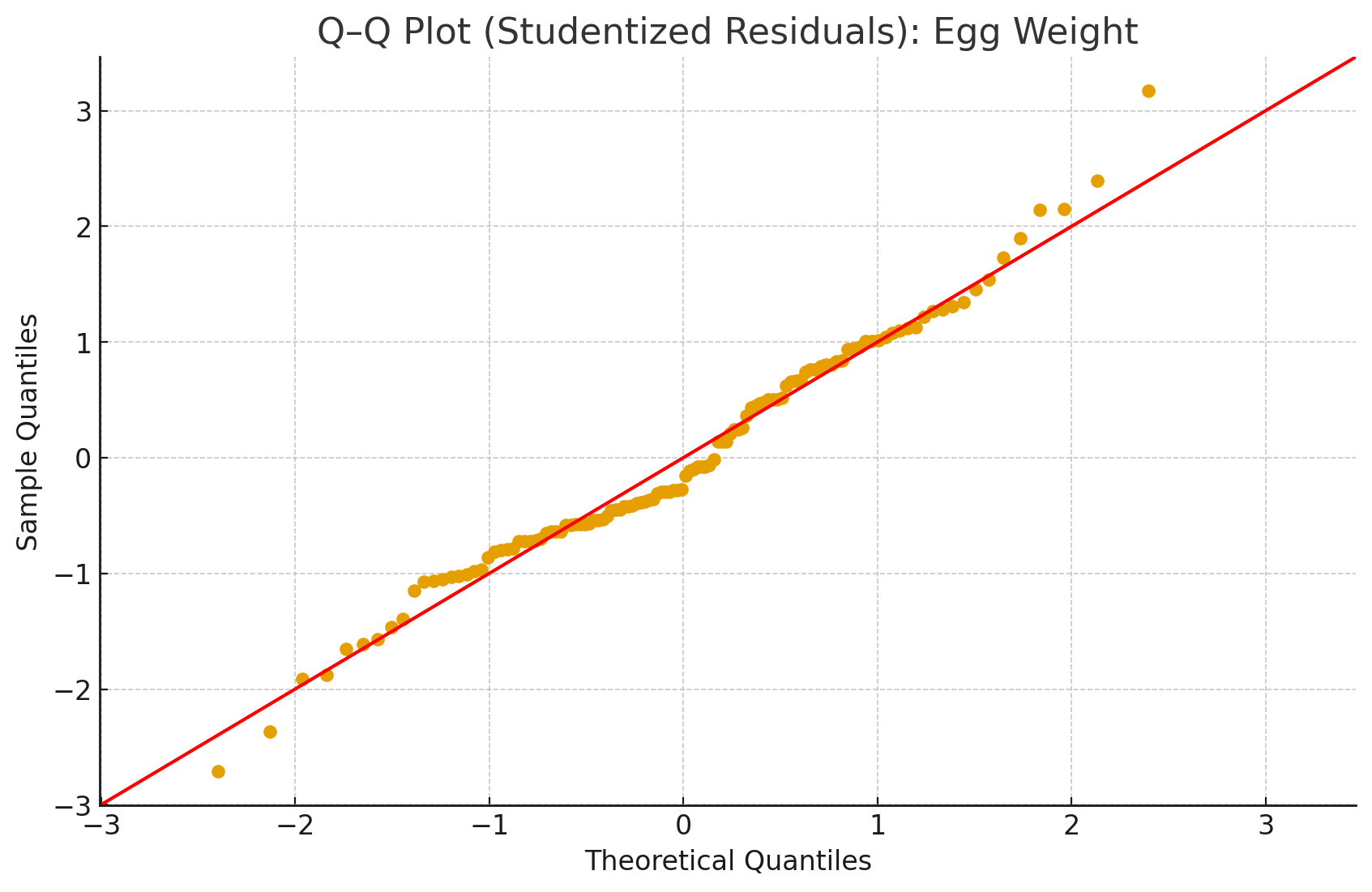


Fig. S2. Q–Q plots of studentized residuals for each trait shown in Table 2.
